# A Nervous Origin for Fish Stripes

**DOI:** 10.1371/journal.pgen.1002081

**Published:** 2011-05-19

**Authors:** Robert N. Kelsh, Gregory S. Barsh

**Affiliations:** 1 Department of Biology and Biochemistry and Centre for Regenerative Medicine, University of Bath, Bath, United Kingdom; 2 Departments of Genetics and Pediatrics, Stanford University School of Medicine, Stanford, California, United States of America; The University of North Carolina at Chapel Hill, United States of America

The current excitement about adult stem and progenitor cells is an opportunity for developmental biologists to shed new light on old problems. Where do differentiated cells in the adult come from, and what else can their progenitor cells do? These questions are especially accessible in the zebrafish, where there is a deep experimental and genetic toolbox, and a developmental pathway with a well-defined metamorphic transition from larvae to adult. One of the premier model systems in this premier-league model organism is pigmentation, since pigment cells in fish—including black melanocytes (known as melanophores in fish), yellow xanthophores, and reflective iridophores—are organized into an array of beautiful, accessible, and evolutionarily diverse patterns that have helped motivate their study [1], [2]. Adult zebrafish show a longitudinally striped pigment pattern, distinct from the embryonic one. The adult pigment cells derive primarily from dormant stem cells, not embryonic pigment cells. But the cellular identity and location of these adult pigment cell progenitors has remained mysterious.

In this issue of *PLoS Genetics*, Budi et al. [3] provide an answer to the cryptic origin of adult pigment cells, concluding that they originate from cells associated with the peripheral nervous system (Figure 1). The findings of this study are remarkably similar to recent work on the developmental origin of mammalian melanocytes [4], demonstrating (once again) the conservation of developmental principles across large evolutionary distances.

**Figure 1:**
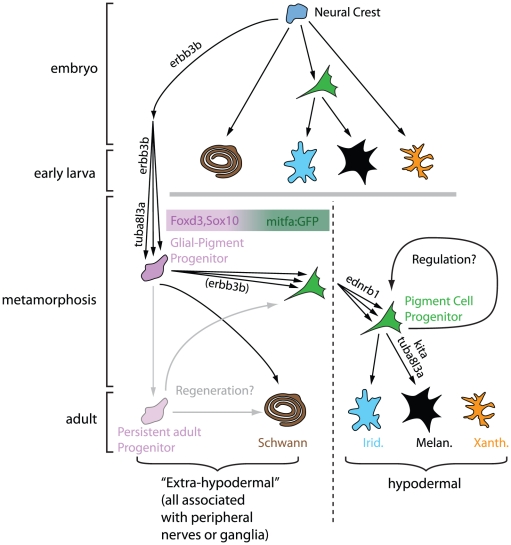
Model for neural origins of metamorphic melanocytes and iridophores. All pigment cells are highly likely to originate from embryonic neural crest, but pigment cells in the embryo and early larva have lineages distinct from pigment cells in the adult. The latter arise from a Glial-Pigment Progenitor population (described as mGP in Budi et al. [3]) that can be recognized in the larval-to-adult transition and exist outside the “skin” in an extra-hypodermal location, identified as within the peripheral nervous system. Once in the “skin” (the hypodermis), the Pigment Cell Progenitor population can further expand and/or differentiate into iridophores (Irid.) and into melanocytes (Melan.). Adult xanthophores (Xanth.) are derived from a different lineage. The regenerative capacity for pigment cells and glia is likely to be explained by the persistence of Glial-Pigment Progenitor cells into adulthood. Modified from Budi et al. [3].

## Cryptic, but Now Revealed

Budi et al. begin their studies by showing that cells that express *foxd3* and *sox10*, markers of both glial and early neural crest cells [5]–[7], are seen in mid-metamorphic stage larvae, in dorsal root ganglia, myotomes, and the base of the dorsal fin. They define these cells as “extra-hypodermal”, since all are outside of the skin, where adult pigment cells are normally found. They noticed that a transgene, *mitfa::GFP*, which in embryos marks pigment cell precursors and differentiating melanocytes [8], is expressed by numerous extra-hypodermal cells, but not before metamorphic stages. Double-labelling experiments suggest that *mitfa::GFP* cells derive from *foxd3-* and *sox10-*expressing cells and that some cells expressing these markers become proliferative prior to and during early metamorphosis. All these progenitor cells are strongly associated with peripheral nerves. Crucially, DiI labelling and time-lapse analysis of *mitfa::GFP* fish demonstrate that a proportion of labelled cells move into the skin, where some differentiate into melanocytes or iridophores.

Further support for the authors' model came from exquisitely detailed quantitative comparisons of progenitor behaviour in key patterning mutants. Many fewer *mitfa::GFP* progenitors arrive in the skin in *erbb3b* and *tuba813a* mutants, which are known to be required for formation of melanocyte progenitors [9], [10]; once there, the mutant cells show abnormalities of differentiation. Previous studies have distinguished early (Kit-dependent) and late (Csfr1- and Ednrb1-dependent) melanophores, differing in where they form and how they contribute to adult stripes [11]–[14]. The authors also show differences in proliferative and differentiation behaviour of *mitfa::GFP* progenitors in mutants for these genes. To what extent these differences can explain the distinctive phenotypes remains to be established, and will likely require detailed mathematical modelling of the patterning process. Nevertheless, this study provides an unprecedented amount of quantitative data on which to base modelling efforts.

An interesting observation emerging from this work comes in particular from the *erbb3b* (also known as *picasso*) mutant studies. Although this mutation severely reduces the number of pigment cell precursors (as defined by expression of *mitfa::GFP*) during metamorphosis, the few mutant cells that do survive to reach the hypodermis actually exhibit a greater degree of proliferation and differentiation relative to wild type. This observation seems to demonstrate the often assumed, but rarely shown, regulative behaviour of pigment cells in vivo.

Finally, the fish's ability to regenerate pigment cells is another fascinating aspect of this model system [15]. It is intriguing that Budi et al. show *foxd3* or *mitfa::GFP-*expressing extra-hypodermal cells persisting in adults since this suggests that they may contribute to regeneration of melanocytes. They use an elegant genetic approach to show that adults have limited regenerative capacity for pigment cells, at least in the double-mutant background used. Although not assessed here, it would be interesting to examine whether extra-hypodermal progenitor cell numbers become correspondingly reduced during this experiment.

## Answers and More Questions

This study provides compelling evidence for a neural origin of adult pigment cells and reveals the *mitfa::GFP* transgene as an invaluable marker for the early stages of their development. These breakthroughs inevitably suggest new questions. Are these progenitor cells neural crest-derived? While overwhelmingly likely, this still needs to be formally shown. Do all adult pigment cells arise from these progenitor cells? The authors have not been able to identify the origin of xanthophores, and the possibility that some melanophores and iridophores derive from other cells remains. Where are the pigment cell stem cells? The authors show that progenitors for *mitfa::GFP* cells express *foxd3* and *sox10* and reside within the peripheral nervous system. This may suggest a dispersed, axon-associated source of pigment cell progenitors. However, these latter cells themselves may arise from spatially more localized stem cells, perhaps in ganglia or specific niches of the nerves, which simply use the peripheral nerves as convenient tramways for dispersal. Even if the stem cells are dispersed and axon-associated, what do they look like? Do differentiated satellite glia and/or Schwann cells have latent potency to form pigment cells? Or are there distinct, dormant stem cells intermingled with them? For now, a Schwann cell precursor, as in mammals [4], is the most parsimonious hypothesis, since both Sox10 and Foxd3 are involved in maintaining multipotency [16]–[18] and are expressed in Schwann cell precursors [5], [6]. Answering these questions will require intensive lineage-tracing studies of single cells at different stages prior to and during metamorphosis.

The progenitor populations identified here are abundant during metamorphosis, and it is far from clear that all will form pigment cells. While many may remain as a source of regenerative cells, what cell types can they form? The authors are understandably cautious, but propose that the *foxd3/sox10*-expressing cells are tripotent (glia/melanocyte/iridophore), while the *mitfa::GFP-*expressing cells may be bipotent (melanophore/iridophore), although separate unipotent progenitors for each cell type are not yet ruled out. Detailed lineage studies will address these issues, and may reveal other, unexpected, derivatives.

This landmark study provides compelling evidence for the location and identity of a cellular source of fish adult pigment cells. We can now anticipate comprehensive examination of these cells in the many available mutants, promising mechanistic insight into the enduring problem of adult pigment pattern formation. Beyond this, the identification of the cryptic progenitors of adult pigment cells should make the fish pigmentary system another tractable model in which to explore general principles of adult stem cell biology. For example, issues such as how stem cell potency is controlled and develops, how multiple cell types are generated in appropriate numbers from them, and how these cells are activated during regeneration can all be explored with this system. It is now just a matter of time before fish pigmentation helps colour our understanding of adult stem cell biology!
